# Eat4Genes: a bioinformatic rational gene targeting app and prototype model for improving human health

**DOI:** 10.3389/fnut.2023.1196520

**Published:** 2023-05-26

**Authors:** Morgan L. Ford, Jessica M. Cooley, Veda Sripada, Zhengwen Xu, John S. Erickson, Kristin P. Bennett, Dana R. Crawford

**Affiliations:** ^1^Department of Mathematical Sciences, Rensselaer Polytechnic Institute, Troy, NY, United States; ^2^Department of Immunology and Microbial Disease, Albany Medical College, Albany, NY, United States; ^3^Rensselaer Institute for Data Exploration and Applications, Renssalaer Polytechnic Institute, Troy, NY, United States

**Keywords:** healthy dietary agents, dietary guide, dietary rational gene targeting, bioinformatics, gene expression, risk genes, app, healthcare

## Abstract

**Introduction and aims:**

Dietary Rational Gene Targeting (DRGT) is a therapeutic dietary strategy that uses healthy dietary agents to modulate the expression of disease-causing genes back toward the normal. Here we use the DRGT approach to (1) identify human studies assessing gene expression after ingestion of healthy dietary agents with an emphasis on whole foods, and (2) use this data to construct an online dietary guide app prototype toward eventually aiding patients, healthcare providers, community and researchers in treating and preventing numerous health conditions.

**Methods:**

We used the keywords “human”, “gene expression” and separately, 51 different dietary agents with reported health benefits to search GEO, PubMed, Google Scholar, Clinical trials, Cochrane library, and EMBL-EBI databases for related studies. Studies meeting qualifying criteria were assessed for gene modulations. The R-Shiny platform was utilized to construct an interactive app called “Eat4Genes”.

**Results:**

Fifty-one human ingestion studies (37 whole food related) and 96 key risk genes were identified. Human gene expression studies were found for 18 of 41 searched whole foods or extracts. App construction included the option to select either specific conditions/diseases or genes followed by food guide suggestions, key target genes, data sources and links, dietary suggestion rankings, bar chart or bubble chart visualization, optional full report, and nutrient categories. We also present user scenarios from physician and researcher perspectives.

**Conclusion:**

In conclusion, an interactive dietary guide app prototype has been constructed as a first step towards eventually translating our DRGT strategy into an innovative, low-cost, healthy, and readily translatable public resource to improve health.

## Graphical abstract

Summary of Eat4Genes and its utility. Using our Dietary Rational Gene Targeting approach as a guide, bioinformatic mining of public databases was carried out to identify human studies assessing gene expression after ingestion of healthy dietary agents with an emphasis on whole foods. This information was used in the construction of the dietary Eat4Genes app, which is able to identify healthy dietary agents able to modulate the expression of key risk genes back toward the normal to potentially treat various health conditions. This app is presently a prototype model that provides dietary suggestions, an important step toward future studies testing these suggestions in clinical trials toward moving the Eat4Gene app to a dietary recommendation resource.
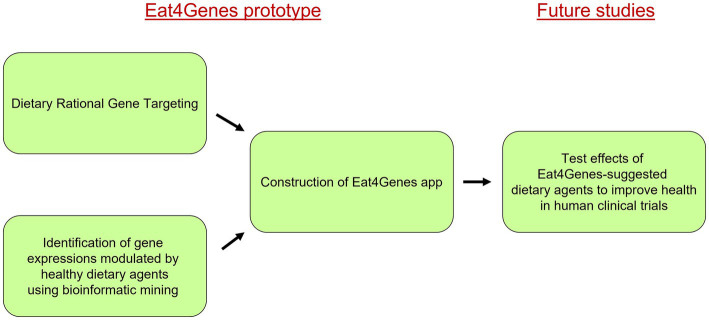

## 1. Introduction

Much of the world’s population has one or more chronic diseases leading to suffering and high costs of health care (1). While pharmaceutical drugs offer valuable treatments, they can also be expensive and associated with toxic side effects in both the short and long term (2). In fact, healthcare costs (per GDP) have more than tripled in the last 60 years (3). Healthy dietary approaches represent a protective and complementary option for such treatments due to their vastly reduced costs and documented health benefits.

We have developed a low-cost and healthy treatment strategy for a wide range of health associated conditions that we call “dietary rational gene targeting” (DRGT) (4–7). Here, healthy dietary agents such as berries, nuts and whole grains are used to modulate disease-contributing gene expression back toward the normal. This strategy offers many advantages over just pharmaceutical drugs in slowing the disease process including lowering treatment cost, reducing drug toxicity, and being readily translatable since it requires achievable diet modification. It represents an important extension of so-called “nutrigenomic” studies with hopes of expanding and accelerating DRGT use for public therapeutic benefit.

Numerous studies have reported beneficial effects from a plant-based diet, with key bioactive compounds such as polyphenols singled out for their health benefits and in some cases, modulation of gene expression (8–12). This has developed into the precision nutrition field maximizing individual dietary benefit as opposed to less effective general population guidelines (13). Precision nutrition is highly complex and considers genetics, epigenetics, microbiota, physiology and much more with a lot still unknown. We feel that dietary rational gene targeting “leapfrogs” much of this highly complicating variability by assessing the levels of individual mRNA (and resultant protein) expressions, which are key physiological effectors, in turn efficiently contributing to a potential individualized precision nutrition dietary strategy. For example, healthy dietary reduction in the expression level of *VEGF-A* (Vascular endothelial growth factor A) to combat cancer metastasis is a good example of our dietary strategy as opposed to only the use of a pharmaceutical drug such as which is expensive and sometimes toxic (14).

While the targeting of key disease-related genes/proteins with healthy diet has excellent promise, a surprising lack of such studies directly translatable to human *in vivo* have been carried out. Instead, most studies have focused on cell culture at non-physiological concentrations of dietary agents and dietary rodent studies that do not translate well to human. Recent studies by Martin-Hernandez et al. (15) and Zheng et al. (16) have provided a valuable framework for a nutrigenomics web app model although this model is limited by its emphasis on purified bioactive compounds in cell culture studies. Cell culture studies are a weak substitute for *in vivo*, especially for dietary studies given the complexity of the human digestive system. Additionally, while some such isolated botanical compounds are available as dietary supplements, their health benefits are unclear due to low bioavailability and limited food matrix synergy (17–21).

Here, we provide a major extension of such studies to emphasize whole food and extracts, *in vivo* human ingestion, and the therapeutic targeting of a wide range of health conditions to greatly improve the potential effectiveness of our food suggestions. Our overall hypothesis is that identification of healthy whole foods that modulate health condition-causing gene expression back toward the normal is a low-cost, healthy, and readily-translatable complementary therapeutic approach to costly and sometimes toxic pharmaceutical drugs. Here “Condition” is defined as any state of health; i.e., optimal or sub-optimal, with sub-optimal then including diseases and disorders. Construction of our new interactive dietary guide app (“Eat4Genes”) examines, for the first time, the relationship between conditions, risk gene expression, and whole food diet in human following ingestion using studies collected from published clinical trials and bioinformatic resources and includes development of a preliminary ranking system for presentation of DRGT results. We envision this prototype app as a key first step toward eventually aiding patients, healthcare providers, community and researchers in treating and preventing numerous health conditions.

## 2. Materials and methods

### 2.1. Bioinformatic mining and *in vivo* dataset

To assess the effect of dietary agents (whole foods, food extracts, and purified phytochemicals) on gene expression, we carried out extensive public database mining. Here, we used the Boolean search operator AND in combination with the keywords “human,” “gene expression”, and separately, each individual dietary agent of interest (below). Databases searched included the National Center for Biotechnology Information’s (NCBI) Gene Expression Omnibus (GEO) (22); PubMed (23); Google Scholar (24); clinical trials published through the U.S. Library of Medicine (25); the Cochrane library (26); and the European Molecular Biology Laboratory’s European Bioinformatics Institute (EMBL-EBI) (27). Where necessary, the NCBI GEO2R web tool was used to analyze noncurated data (28). Hits were further filtered to select only studies involving human oral consumption of these dietary agents, appropriate controls, and clearly accessible and interpretable data. Retrospective questionnaire studies were excluded.

### 2.2. Dietary agents searched (keywords)

As part of the above databases searches, each of the following dietary agents were searched representing whole foods, whole food extracts, and purified phytochemicals with reported health benefits: grape, olive oil, ginkgo, soy, oatmeal, grains, cereal, garlic, blueberry, walnuts, cashews, pistachios, hazelnuts, Brazil nuts, almonds, broccoli, kale, spinach, ginger, mushrooms, pineapple, nuts, beans, ginseng, pomegranate, legumes, kiwi, flaxseed, acai, black cherry, rosemary, mango, oranges, apples, passionfruit, coffee, yogurt, fiber, green tea, vegetable juice, turmeric, epigallocatechin gallate (EGCG), curcumin, genistein, quercetin, resveratrol, lycopene, berberine, hesperidin, sulforaphane, and apigenin. These dietary agents were selected based on significant published evidence for their health benefits (e.g., 8-12, 20-21, 29-43) with the caveat that there is no known perfectly healthy food.

### 2.3. DRGT data collection, cleaning, and preparation

The DRGT dataset at the heart of Eat4Genes has three main data sources: our compiled list of *health conditions* and the desired *risk gene* modulation to improve these conditions (condition-to-gene abbreviated “condition2Gene”); our compiled curated list of human *in vivo* ingestion *studies* that quantify how consumption of *nutrients* influences gene expression (“geneData”); and *in vitro* human cell culture results available from NutrigenomeDB.

**Figure 1:**
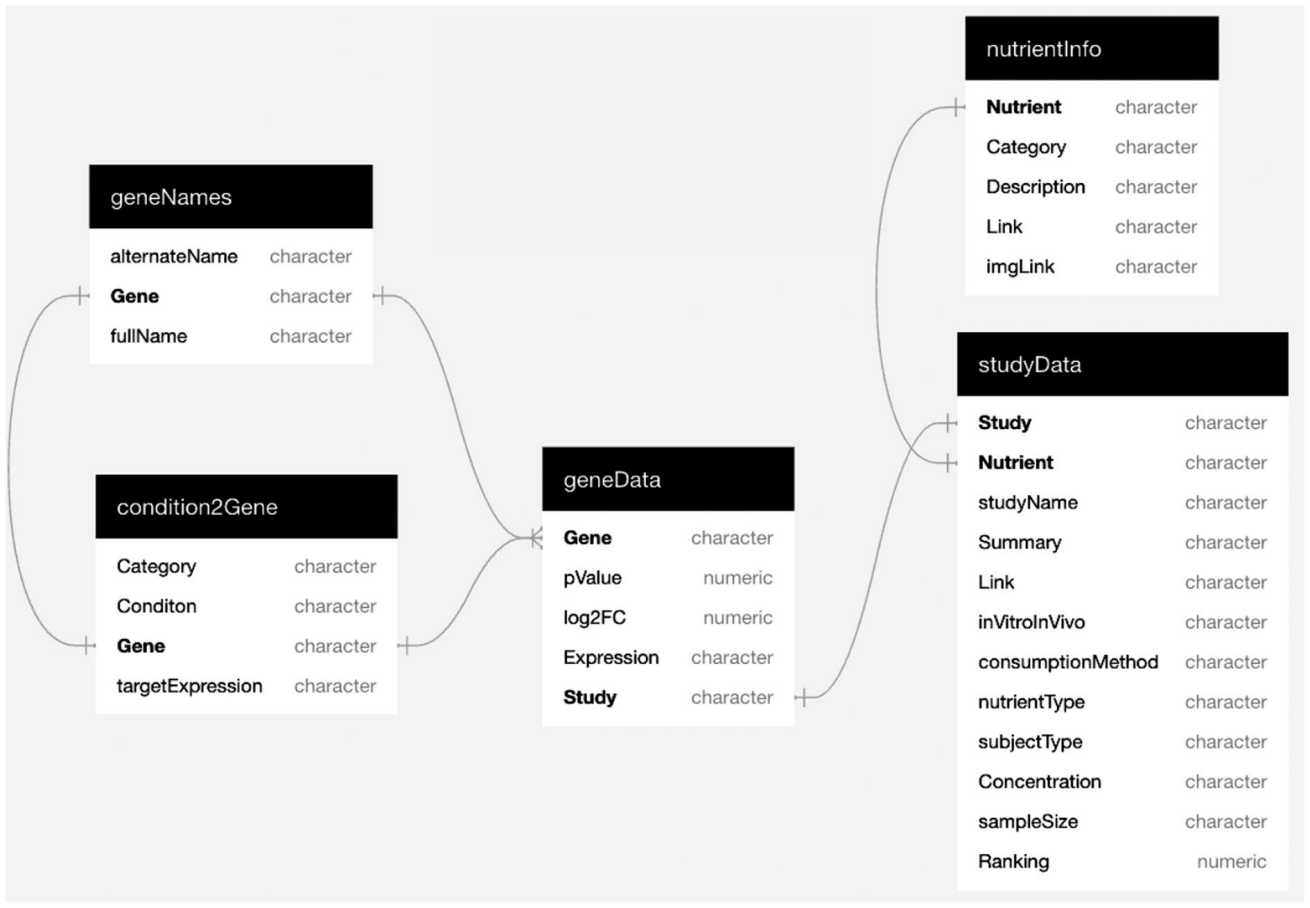
Eat4Genes Dietary Rational Gene Targeting (DRGT) dataset. The Eat4Genes DRGT dataset consists of these five linked sub tables. Fields designated as characters and numerics are strings and numbers, respectively.

The **condition2Gene** subtable was created to link health conditions to known key risk genes. Such risk genes were identified through independent compilations from PubMed, known pharmaceutical targets, and the NCBI resource “Genes and Disease” (29). The **geneData** subtable contains the data for all of the mined dietary gene expression studies. The gene expression data was extracted from the studies and converted to log_2_-derived fold change (L2FC). While all results were included such as no (neutral) gene expression change, our preferred and highest rated data are dietary conditions leading to a greater than 1.2-fold (L2FC > 0.263) up-or down-modulation (a value within the wide cutoff range of our references), and with a normal value of *p* of <0.05. In addition to the above, a “**geneNames**” subtable was created to form a consistent naming convention using the HGNC (HUGO Gene Nomenclature Committee) site multi-symbol checker tool to find approved gene symbols and full gene names. The subtable **nutrientInfo** contains information on the nutrients. We use the word “nutrients” to include healthy phytochemicals as well as functional foods (e.g., fruits and vegetables) that can be consumed by humans. The dataset contains 51 different nutrients and each is linked to the category (whole food, whole food extract, or phytochemical), a brief description, a link to an external webpage with more information, and a link to a small icon to represent the nutrient. Finally, the **studyData** subtable includes the reference name of the study; the nutrient; the full name of the study; a brief summary; the GEO accession number used for the expression data (if available); a link to the study; whether the study was *in vivo* or *in vitro*; the consumption method; the type of subject; the concentration of the nutrient used; the sample size of the study; and the ranking. Combined, the Eat4Genes DRGT dataset consists of these five linked subtables that capture the relationships between nutrients, scientific studies, conditions, genes, and gene expression (Figure 1).

In addition to our human dietary ingestion mining data, part of this dataset was derived from the NutrigenomeDB web resource (15). While NutrigenomeDB content is almost all *in vitro* and so significantly less valuable, it is still of some interest to our dataset. Here, we cloned the NutrigenomeDB relational database from the publicly-available github repository (30) and exported its nutrigenomic table. The resulting dataset consisted of 48 studies involving two whole foods and 24 nutrients. The NutrigenomeDB team had already performed a differential expression analysis of nutrigenomics experiments performed on human cells found in the Gene Expression Omnibus (GEO) repository. Therefore, we were able to incorporate these results directly into the below Eat4Genes app.

### 2.4. Eat4Genes app construction

We harmonized and joined the above data and embedded it in the Eat4Genes web app using Shiny (31), a framework for easily implementing web applications based entirely on R [20] and allowing users to interactively query and visualize data. It is modeled as an interactive webpage that serves as a food guide to help treat or prevent specific conditions based on the targeting of key genes. The prototype app has been publicly deployed (32) and its source code and DRGT dataset are available *via* a public github repository (33).

The app build strategy included home page construction with the option to select dietary suggestions for either a specific condition or specific gene through drop-down menus. For the “By Condition/Disease” selection, four sub-menus then appear including Food Guide, Targeted Genes, Full Report, and Data Sources. To view results for the “By Gene” selection, two sub-menus are available including Food Guide and Data Sources. For visualizations, bar charts and bubble charts are provided as choices. The package used to create this is *highcharter* (34), an R-based wrapper of the Highcharts (35) Javascript library. Overall, the app is organized so that the main results are provided on the second page that the user sees; as the user advances through sub-pages, higher-level details based on their selections are provided. The main results page also has two different visualizations for the user to choose from as well as text results to accommodate either lay or scientifically-knowledgeable viewers.

### 2.5. App ranking system

The goal of the ranking system is to assess the likelihood that users will potentially achieve a desired risk gene modulation with app selected dietary agents. At this time and at the most basic level, our rankings are based – from highest to lowest – in the order human whole food oral ingestion, whole food extract (such as extra virgin olive oil) oral ingestion, purified dietary supplement oral ingestion, cell culture studies, and cell culture studies with higher, less physiologically-relevant dietary supplement concentration. That is, clinical trials in which humans ingest nutrients and then have their changes in gene expression analyzed are, in general, considered the highest quality studies because their results are most likely to translate into practice. *In vitro* human studies (that is, studies of how nutrients change gene expression in human cell lines) are also considered but do not necessarily translate into *in vivo* effects so are considered of lesser value. In addition, the extent of each fold modulation (effect size) and associated statistical significance also contribute to our rankings, with modulations of *p* < 0.05 given more weight than *p* > 0.05. Independent duplicate studies with the same nutrient demonstrating the same direction of modulation are also rated higher although there are presently a very limited amount of such studies. The heuristics used in the relative rankings are given in github (30) with sufficient detail that they can easily be improved upon and even replaced in future research while preserving all functionality of Eat4Genes. Given the lack of human *in vivo* ingestion studies, we are not able to construct a more meaningful detailed numeric ranking system at this time and thus, consider this above relative ranking as a basic introductory guide. Nonetheless, since this is such an important consideration for translating our dietary guide to human usage, we are establishing this basic relative ranking for our current Eat4Genes app prototype with the caveat that it can be expanded upon over time (See Discussion).

### 2.6. Statistical analyses

Effect sizes and statistical analyses were obtained from each mined source; i.e., these gene expression datasets were directly downloaded from each study. Experimental designs varied and included microarray, qPCR and RNA sequencing but were uniformly reported as normalized data using reference controls. For our proposed prototype ranking system for presentation of DRGT results, these data were sorted by effect sizes and correlate *p* values presented without filtering, with higher and lower values, respectively, prioritized. An effect size versus *p* value plot is also included in the app Full Report to highlight the most prominent and significant gene expression modulations. Cohort sizes and time of test diet administration for each study are presented in both tables and underscore the wide range of experimental designs.

## 3. Results

### 3.1. Bioinformatic mining

Fifty-one human healthy dietary agent ingestion studies were identified in the examined databases that met our qualifying criteria. Of these, 37 were human whole food or whole food extract studies as listed in Table 1 (36–70). These include 18 different whole foods and extracts (out of the 41 whole food items searched). Some of these studies included multiple timepoints, increasing the usable data. GEO and/or PubMed databases contained all such data; i.e., this data was not scattered throughout the various databases utilized. Despite varying conditions (e.g., time of test diet administration), the number of modulated genes for those studies examining whole genome expression was reasonably consistent (1,061 ± 227 SEM). Whole food human ingestion studies reported to modulate genes included grape, Brazil nuts, walnuts, hazelnuts, pistachios, soy, broccoli, yogurt, blueberries, bilberries, garlic, kiwi, and fruit and vegetable blend concentrate (36, 39, 42–47, 49, 51–55, 64–67). Whole food extract human ingestion studies reported to modulate genes included grape extract, green tea extract, soy extract, olive oil, broccoli extract, orange juice, Bergeris vulgaris juice, and apple and blueberry juice (37, 38, 40, 41, 43, 48, 50, 56–63, 68–70). In addition, purified bioactive phytochemicals with gene modulatory effects were identified from 14 independent studies (63, 71–83). They included curcumin, quercetin, resveratrol, EGCG (epigallocatechin gallate), genistein, soy isoflavones (genistein, daidzein, glycitein), DIM (3,3′-diindolylmethane), sulforaphane, and hesperidin (Table 2). Modulated gene expression was observed in a variety of cells/tissues in these studies including peripheral blood mononuclear cells (PBMCs), white blood cells, whole blood, blood monocytes, breast cancer biopsies, prostate cancer biopsies, nipple aspirate fluid, rectal biopsies, muscle biopsies, nasal lavage cells, blood lymphocytes, and bronchoscopy cells. In addition, we identified 96 key risk genes for a wide range of pathologies (e.g., *HMGCR* for Hypercholesterolemia) for our app.

**Table 1:** Human whole food or whole food extract ingestion gene expression studies.

Food type	Diet time	Cell/tissue	*N*	References
Grapes	3 weeks	PBMCs	6	(36)
	1,2,4,12 h	blood lymphocytes	6	(37)
	8 weeks	whole blood	13	(38)
	3 h and 4 weeks	PBMCs	20	(39)
	6–12 months	PBMCs	6	(40)
	8 weeks	muscle biopsies	38	(41)
	9 weeks	whole blood	10	(42)
Nuts	6 weeks	rectal biopsies	9	(43)
	2–3 weeks	breast cancer/normal biopsy	5	(44)
	4 weeks	whole blood	22	(45)
	6 weeks	whole blood	12	(46)
	4 months	pistachios	49	(47)
Soy	7–30 days	breast cancer biopsies	51	(48)
	8 weeks	PBMCs	18	(49)
Broccoli	4–8 weeks	prostate biopsies	48	(50)
	3 days	nasal lavage cells	5	(51)
Yogurt	9 weeks	PBMCs	30	(52)
	2,4,6 h	whole blood	6	(53)
	2,4,6 h	whole blood	7	(54)
	8 weeks	PBMCs	25	(55)
Olive oil	4 h	PBMCs	12	(56)
	4 h	PBMCs	12	(56)
	3 weeks	PBMCs	18	(57)
	3 weeks	PBMCs	18	(58)
	5 h	PBMCs	13	(59)
	6 h	PBMCs	6	(60)
	3 weeks	PBMCs	10	(61)
	4 h	PBMCs	20	(62)
Other				
Orange juice	4 weeks	white blood cells	10	(63)
Blueberries	4 weeks	PBMCs	40	(64)
Bilberries	8 weeks	PBMCs	27	(65)
Garlic	3 h	whole blood	17	(66)
Green tea extract	6 weeks	rectal biopsies	11	(43)
Kiwi	8 weeks	whole blood	10	(67)
Berberis juice	8 weeks	whole blood	40	(68)
Fruit/veg juice	8 weeks	whole blood	28	(69)
Apple juice	4 weeks	lymphocytes	143	(70)

PBMCs, peripheral blood mononuclear cells; *N* refers to the study participant number.

**Table 2:** Human purified phytochemical ingestion gene expression studies.

Dietary agent	Diet time	Cell/tissue	*N*	References
Curcumin	0–12 h	white blood cells	12	(71)
DIM	4–6 weeks	white blood cells	13	(72)
EGCG/RES	12 weeks	SAT abdominal sub-Q	25	(73)
Genistein	3–6 weeks	prostate biopsies	10	(74)
	84 days	PBMCs	20	(75)
	1 month	prostate tissue	14	(76)
Hesperidin	4 weeks	white blood cells.	10	(63)
Isoflavones	6 months	nipple aspirate	49	(77)
Quercetin	2 weeks	blood monocytes	3	(78)
	12 weeks	PBMCs	78	(79)
Resveratrol	12 weeks	adipose/skeletal tissue	15	(80)
	30 days	muscle	10	(81)
	2 months	PBMCs	46	(82)
Sulforaphane	6 weeks	bronchoscopy cells	29	(83)

EGCG, epigallocatechin gallate; DIM, 3,3′-diindolylmethane; RES, resveratrol; PBMCs, peripheral blood mononuclear cells; *N* refers to the study participant number.

### 3.2. Bioinformatic mining: *in vivo* versus *in vitro* and ranking

Although these dietary human ingestion studies were enough to construct our below Eat4Genes app, our mining also revealed a surprising modest number of studies translatable to human *in vivo* prior to filtering, underscoring the need for more such studies. Instead, beyond the Table 1 whole food-related studies, most dietary related gene expression studies found used human cells in culture as are well summarized in NutrigenomeDB (15), many at non-physiological concentrations of dietary agents. Even the above 14 human *in vivo* ingestion studies with purified phytonutrient supplements are of more questionable value given their typical poor bioavailability *in vivo*, their uncertain long-term effects as compared with whole foods, and their high concentrations in some cases. Based on this, we prioritized our mined studies as detailed in the Methods “Ranking” section. Exact rankings are not possible at this time given the lack of *in vivo* data such as confirmatory studies by independent laboratories carried out under similar conditions. Nonetheless, our “relative” ranking system provides a prototype foundation for an important DRGT issue, and one that will be improved and refined over time as additional clinical studies are performed.

### 3.3. Eat4Genes app implementation

**Figure 2:**
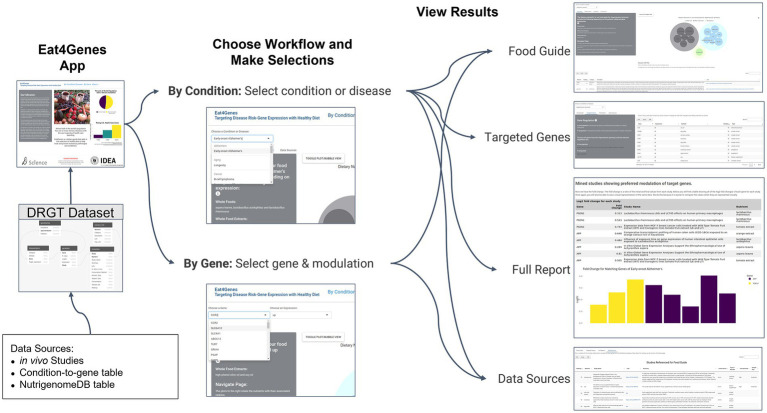
Eat4Genes App Flow. DRGT data are used as the basis for the DRGT Dataset that is the foundation of the Eat4Genes App followed by choosing a workflow, selecting a condition or gene and modulation, and viewing results. All app selections shown are illustrated using thumbnails.

Figure 2 shows the basic structure of Eat4Genes. Users first enter a home page that describes the app, then obtain nutrient suggestions either for a condition by selecting *By Condition/Disease* or for a specific gene by selecting *By Gene*. Subsequent results are presented in four views: *Food Guide*, *Targeted Genes*, *Full Report*, and *Data Sources*. In summary:

- Home Page: the user first sees the Home page. The left panel contains our mission statement, which is also the introduction to this paper. The right column of the home page presents some statistical evidence for the value of our food guide.
- By Condition Page: users can search for results based on a specified condition or disease selected through a drop-down menu. Results will then show all of the nutrients that modulate the expression of key risk genes and in a direction that benefits a particular condition. This page has four sub-menus: Food Guide, Targeted Genes, Full Report, and Data Sources. In summary:

*Food Guide Page*: the left panel of the Food Guide page shows the dietary nutrients suggested for each health condition classified by category. Beneath that, there are instructions on how to navigate the page and a brief explanation of the rankings. On the right side, the user is shown the visualization model with interaction instructions. Underneath the main section of the page, the user can view more information on the food guide in table form, including the nutrient, the ranking, the category, a brief description, and a link to an external webpage.

*Targeted Genes Page*: the main result of the Targeted Genes page is a table showing the matches of key risk genes for the condition. It contains, for each matching gene: the gene, the direction of modulation, the nutrient, the ranking, and the type. On the left side of the page, a panel shows the user’s information about the table. It explains up and down regulation of gene expression. It also shows the user the key risk genes found in the Eat4Genes database for the condition they have selected.

*Full Report Page*: the Full Report provides a summary of the data given to the user on the app in more detail, intended for healthcare providers and others interested in a more in-depth review of the data pooled to create the food guide.

*Data Sources Page*: this page provides the user with the studies referenced for the food guide that they have received. Here the user can see, for each study: the ranking, the nutrient, the name of the study, a link to the study, a brief summary, whether the study was *in vitro* or *in vivo*, the type of nutrient, the concentration of the nutrient used in the study, and the sample size of the study.

- By Gene Page: the users have the option to search for results by specifying a gene and desired expression direction: up or down. The results show only the nutrients that affect that gene’s expression in that specified direction. This page has two sub-menus: food Guide and Data Sources. These pages have the same above layouts as their counterparts in the By Condition page.
- About Page: this page provides the user with a summary of the purpose of the Eat4Genes app, an explanation of the ranking system, and our defined food type categories (whole foods, whole food extracts and phytochemicals).

To make results accessible to a wider audience, Eat4Genes provides visualizations of the Food Guide section as bar charts or bubble charts to enable a choice of views. In the bubble chart, the size of each circle is based on the ranking value. To show users the different types of recommended nutrients, the app uses a hierarchical packed bubble graph, which groups the bubbles by category. When the user hovers over a bubble, the name of the nutrient, a small image, the ranking, and a description with important information about the nutrient are shown. When the user clicks on the bubble, they are directed to a website with more nutritional information.

Eat4Genes is also targeted to two different demographics: (1) lay users seeking only basic details, such as less technically-knowledgeable patients or community members, and (2) those seeking more details, such as healthcare providers, technically-knowledgeable patients, and biomedical researchers. This added detail is provided in the optional Full Report menu. It was added based on feedback recommendations from our Usability survey (*N* = 17), which was otherwise positive about the app outlay.

### 3.4. Patient consultation scenario

We illustrate the utility of the app in two usage scenarios within the limitations of our current prototype app as discussed below. In the first scenario, someone diagnosed with hypertension, or a healthcare professional on their behalf, consults the Eat4Genes app to obtain dietary guidance as a complement or alternative to pharmaceuticals. On the app’s home page, two workflow options are presented: search by a condition or search by a specific gene. Since this user is searching by a condition, the *By Condition/Disease* option is selected at the top of the page and the user is prompted to select their health condition.

**Figure 3:**
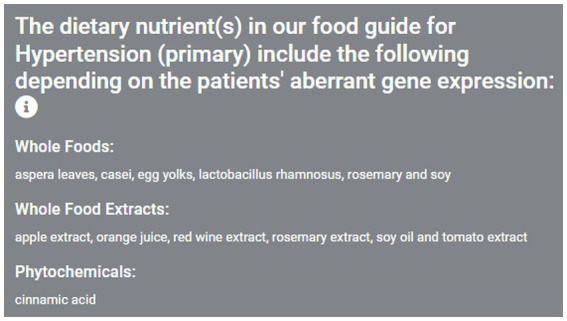
Identification of nutrients found to modulate key risk genes starting from disease selection. As a patient consultation scenario example, someone with hypertension consults the app and first chooses the search By Condition/Disease option. The next page Food Guide sub-menu page then appears showing a list of dietary agents found to modulate the expression of key hypertension-associated risk genes, sorted by category.

**Figure 4:**
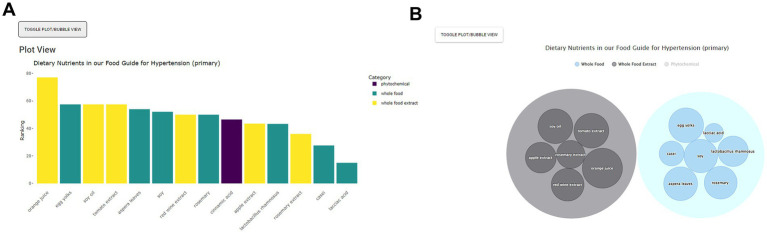
Food Guide bar **(A)** and bubble **(B)** plots showing suggested foods for hypertension along with their rankings and categories. As per Figure 3, the Food Guide sub-menu page first appears after Homepage “By Condition/Disease” selection. The subsequent Food Guide sub-menu view includes right side plots of optional hypertension dietary nutrients. These are viewable as either bar or bubble plots through the toggle view option.

On the next page, the Food Guide sub-menu page first appears and on the left side, the user sees a list of the dietary nutrients sorted by category (Figure 3). On the right-hand side, the user sees a plot of the suggested foods, ordered by ranking, and colored by nutrient category. For this scenario, orange juice, egg yolks, soy, soy oil and rosemary are among the top suggested foods. The user can then download an image file of their results if desired (Figure 4A). At the bottom of this page, a table of results used to create the food plots can be viewed and, in the case of a healthcare professional user, this information used to explain the food guide to their patient.

**Figure 5:**
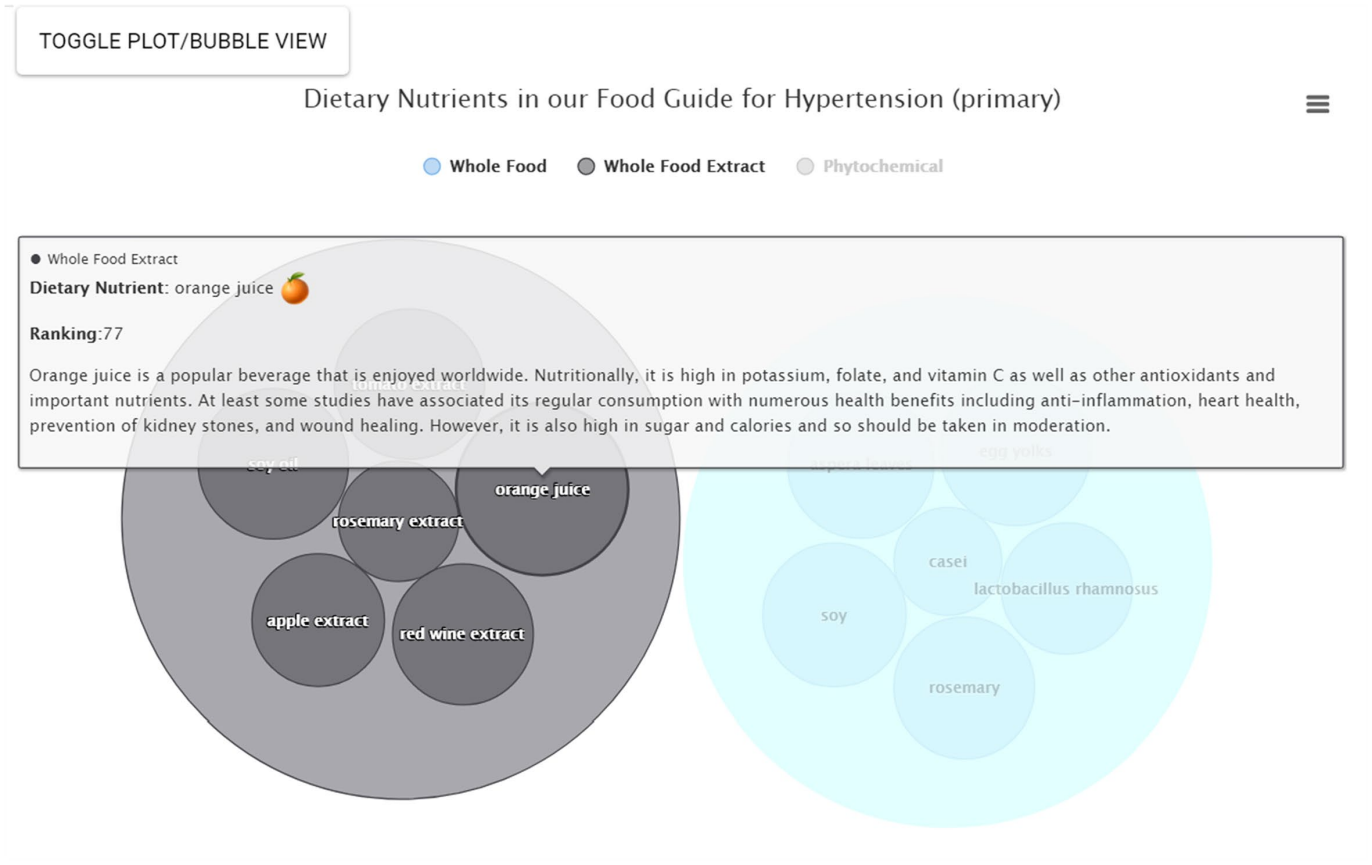
Mouseover information. In the bubble plot view, additional information can be obtained using a mouseover. This information includes nutrient name, representative nutrient image, nutrient category, relative ranking, and a brief description.

For some users the interactive bubble view, annotated with additional information, may be more effective and compelling. From Food Guide, the user simply selects Bubble View to view nutrients in the form of a bubble plot on the right-hand side (Figure 4B). Each category can also be clicked to remove or emphasize; e.g., the “Phytochemicals” category can be clicked to remove it such that only whole foods and whole food extracts are now displayed. Larger bubbles represent the relatively stronger suggestions and the nutrients are sorted by category. The user can also hover over a given nutrient for additional mouseover information; e.g., hovering over orange juice reveals the nutrient name, a small image, the relative ranking, and a brief description (Figure 5). Clicking on the bubble opens a new tab with even more information. All plots can also be downloaded.

**Figure 6:**
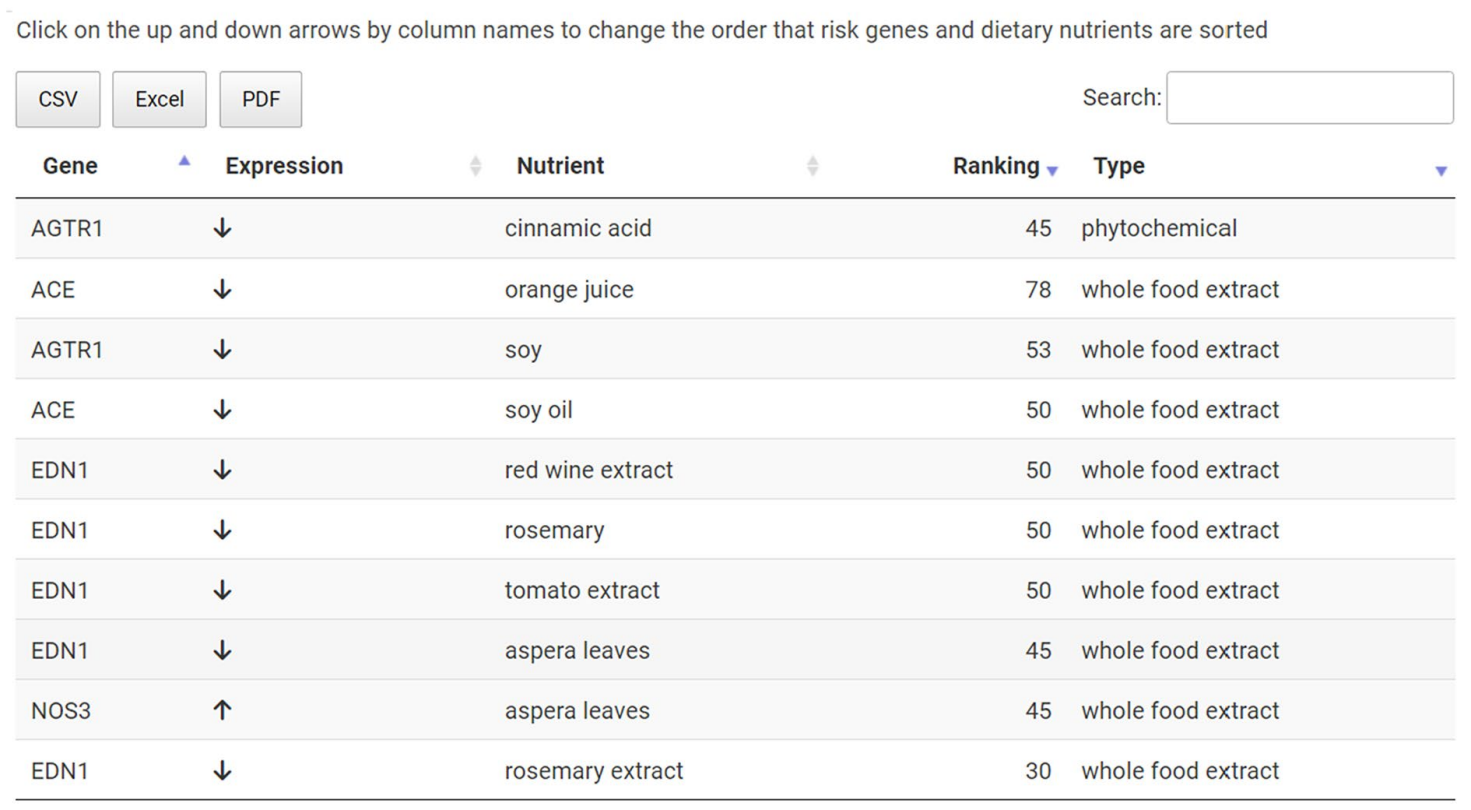
Summary table on the Targeted Genes sub-menu page. Key risk genes for a given disease, in this case hypertension, are listed here along with nutrients that modulate their expression. This information also includes the direction of gene expression modulation, nutrient category and ranking.

**Figure 7:**
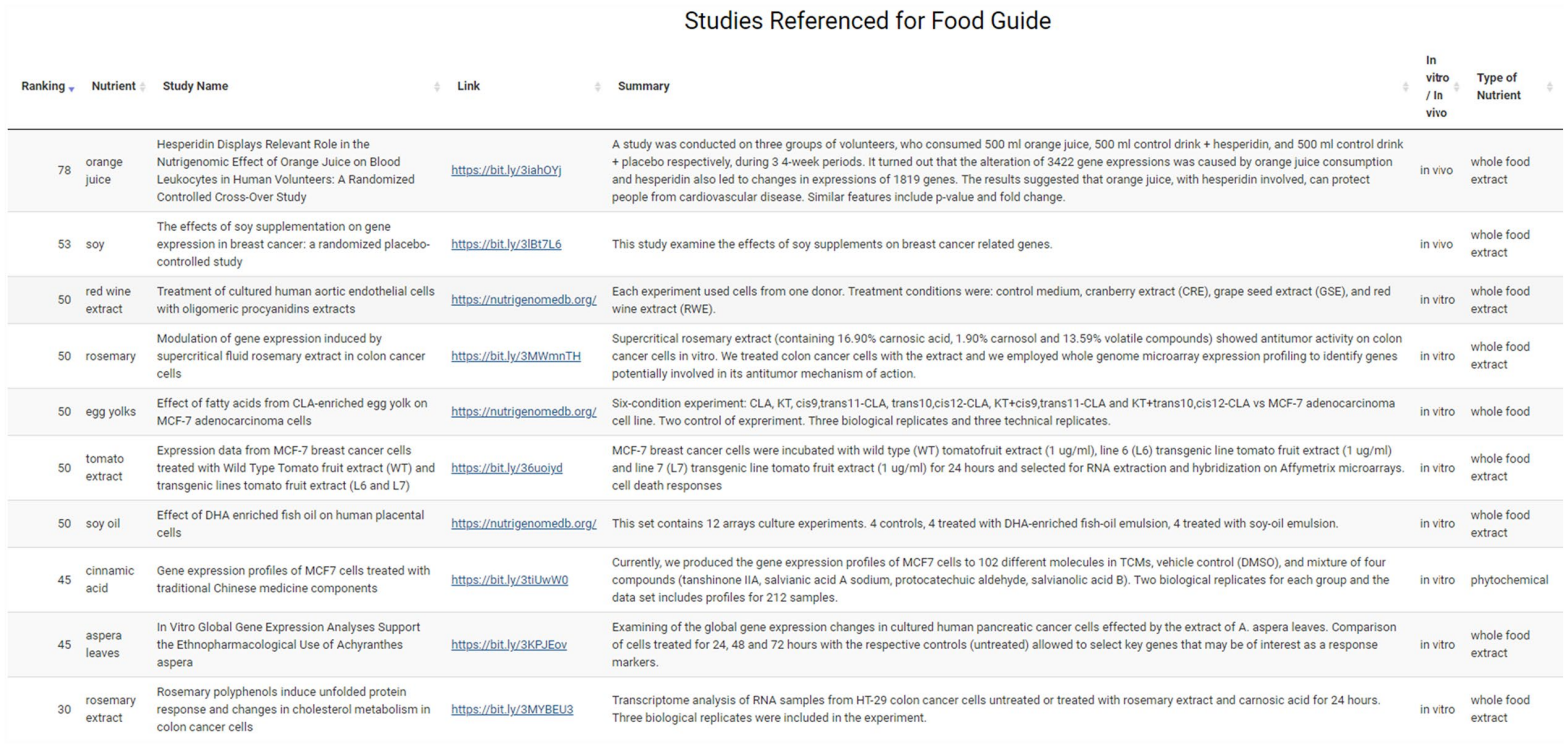
Summary of studies on the Data Source sub-menu page. Information on the research study including links, study name and summary are available on this page, along with modulating nutrient, nutrient category and ranking.

The user can next explore which hypertension-associated key genes were targeted by these foods on the Targeted Genes sub-menu page. Both upregulated gene expressions (e.g., NOS3) and downregulated gene expressions (e.g., ACE) are shown here for this hypertension example with nutrient and ranking information also provided (Figure 6). Finally, the user can access the evidence behind the dietary suggestions by navigating to the Data Sources sub-page. Figure 7 shows the Data Sources table generated for hypertension that includes basic information about each study, the study title, and links to the published paper. Here the provider sees that the results are based on 14 studies, two *in vivo* and twelve *in vitro*. The top *in vivo* study examined orange juice as a potential protective agent for cardiovascular diseases (63), making it more likely to be directly relevant for hypertension. The second *in vivo* study examined gene expression differences caused by soy in patients with breast cancer (48). An eventual judgement would eventually be needed as to whether a study such as this (e.g., using a cancer patient study population) is relevant to address hypertension for the patient being considered. Relative to this, we emphasize that we are describing an app prototype in this manuscript and so throughout, carefully use the word “guide” instead of “recommendation.” The latter word is not yet warranted through use of the current app and will require, among other things, additional confirmational studies by independent labs and determination of how representative gene expression response to a given nutrient for anyone compares to studies of those with existing pathologies such as the above soy example.

### 3.5. Researcher scenario

We now consider a second scenario in which the user is a research scientist studying a particular gene, e.g., the hypertension risk gene ACE (57). This scenario could also apply to patients who know the aberrantly expressed condition gene responsible for their pathology (e.g., ERBB2/Her2 for women with Her2-dependent breast cancer). For a researcher, this could help them consider translationally-relevant human dietary intervention studies, which nutrients to include in these studies relative to their research gene or health condition of interest, and to hypothesize how their app findings could be translated into practice through diet for further study. For ACE and hypertension, they could use the app to determine which nutrients may have a beneficial gene expression modulation (reduction in the case of ACE) to potentially treat hypertension.

**Figure 8:**
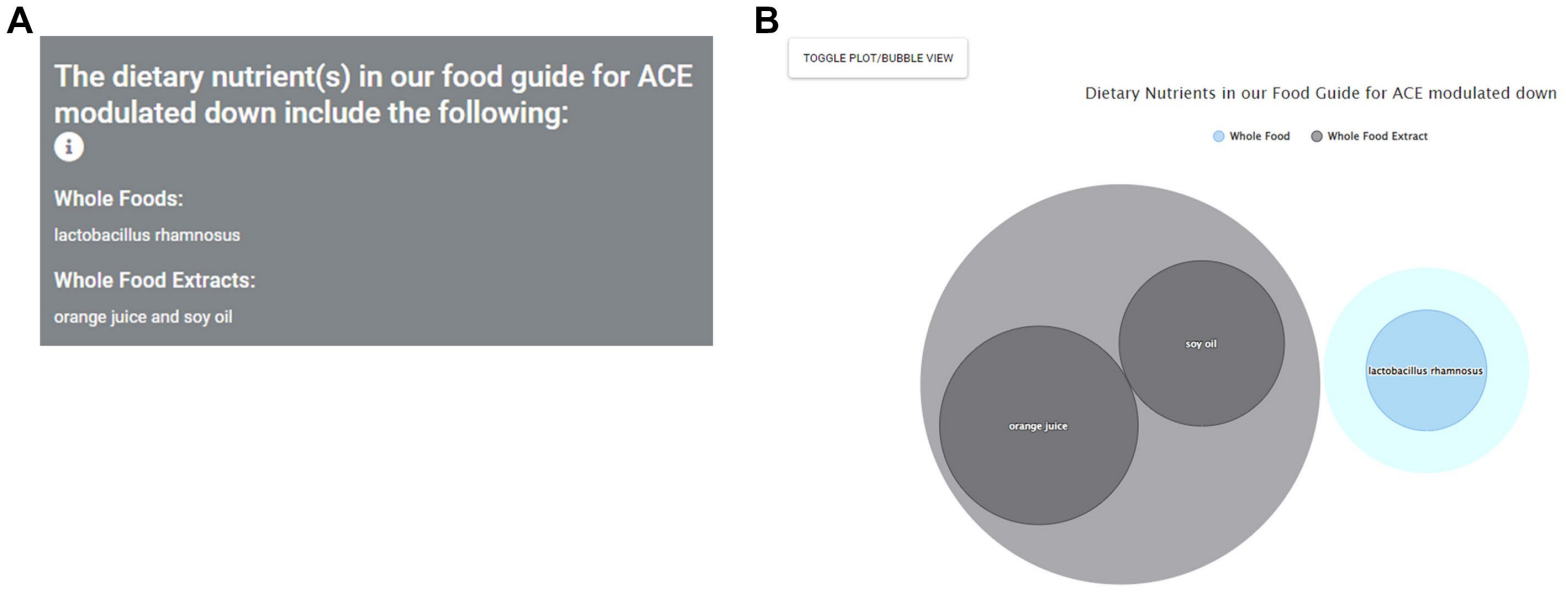
Identification of nutrients found to modulate key risk genes starting from gene selection. As a researcher scenario example, a researcher investigating hypertension and the key risk gene ACE consults the app and first chooses the search By Gene option. ACE can then be input or chosen from the Choose a Gene sub-menu option leading to a listing of dietary agents found to modulate the expression of this gene, sorted by category. Nutrients found to reduce ACE gene expression as desired are then shown as text **(A)** and, in this view, bubble plot **(B)**.

Navigating to the top of the homepage, they would select the By Gene option. The user will then be given the option to search for a specific gene, either by typing it in or scrolling down the list of genes, and can also indicate their desired gene expression modulation (up or down). This in turn leads to the sub-page “Food Guide” again, but this time the results use only the ACE gene to identify healthy dietary agents that down modulate its expression. These results are listed in the panel on the left side of the page (Figure 8). The researcher now sees nutrients associated with down modulation of ACE. In the bubble chart (Figure 8), the researcher can see each nutrient, as well as the strength of evidence for this nutrient, as displayed by both the relative ranking and the size of the nutrient’s bubble. The researcher can also use the toggle button to see results in the plot view. This researcher will likely also want to read the original data studies using the Data Sources sub-page as above for the patient consultation scenario.

## 4. Discussion

Eat4Genes represents a significant first step towards translating the DRGT approach into a clinical deliverable in the form of a dietary guide for healthcare providers, patients, researchers, and the general community. Toward this end, we have carried out a large scale bioinformatic mining of data from multiple public gene expression databases to compile a list of genes modulated by numerous dietary nutraceuticals with a documented history of health benefits. The prototype DRGT dataset is available as an expandable resource for DRGT research and applications. The prototype Eat4Gene app enables users to find dietary guidance by condition or by genes with visualizations that show the findings along with the scientific evidence supporting them.

The prototype Eat4Genes dataset and app serve as a foundation for future work on a dietary treatment guide for patients. It is the first dietary app, to our knowledge, to focus on how whole foods change gene expression beneficially for conditions based on key health condition risk genes supported by rigorous scientific studies. This resource is an extension of the digital web model created by Martin-Hernandez et al. (15) and Zheng et al. (16).

Eat4Genes also provides a basic foundation for dietary suggestion ranking and its use in Eat4Genes. We emphasize that implementation of exact ranking is not possible until a significant number of quality *in vivo* clinical studies are carried out that confirm dietary suggestions, both at the level of target gene modulation and associated health benefit. There are simply not close to this number of available such studies – including replicated studies by independent groups – at this time. Nonetheless, we herein provide a useful strategy for such a ranking system that is a “relative” ranking at this time and based on comparing and prioritizing studies of interest. Thus, our present app numbers are not useful *per se* as absolute indictors of a study’s ranking but rather in comparison with other studies. For example, on a 1–100 scale, a present ranking of 90 means that this study compares well with other available studies based on criteria presented in the methods section. In the future, we envision that a 90 ranking would no longer be a relative assessment but rather a stand-alone value indicating excellence as assessed by numerous criteria, some not even available yet to us. These criteria would include present prioritized categories (i.e., with human whole food ingestion at the top and cell culture studies with high nutrient concentration as the bottom as well as study *p* values, gene modulation effect sizes, and independent lab verification of observed nutrient gene expression modulations) plus future data from many more independent lab verification studies of observed nutrient gene expression modulations; comparison of healthy individual study gene expression results to those where the study subjects have pathologies; comparative dietary agent concentration(s) and quantity range; sample size (already partially addressed by statistical significance analysis); prioritization of greater gene expression modulations; epidemiological study comparisons; and the inclusion of rodent studies although as lower prioritized studies.

In addition to patient/healthcare professional/general community value, Eat4genes also supports scientists interested in translational research. These researchers can refer to our app for identification of healthy dietary agents that modulate their research gene(s) and condition(s) of interest. If such dietary agents are found, and they modulate a given gene of interest in the right direction, they have the potential of providing future therapeutic benefit for patients. This provides a new potential line of research for investigators, such as basic preliminary studies to see if the modulation identified in our app also occurs in a relevant cell culture line or *in vivo* model of interest and, if successful, follow-up clinical-related studies.

It is also important to consider food matrix contributions to any *in vivo* dietary response, whether it’s whole food or purified botanicals. The food matrix refers to the complex physical and chemical interactions of compounds present in food and how this affects bioavailability (5, 18). Thus, whole food effects vary depending upon the mixture of ingested foods. Purified dietary supplement effects and bioavailability would also vary depending on the either synergistic or antagonistic effects of whole foods taken with them, or if they are taken without food. How this impacts our gene expression endpoint will require future controlled studies focused on specific botanical compounds taken in combination with different whole foods.

The main limitation to our approach thus far is as mentioned above: a limited pool of public database human ingestion studies to draw from such that the present app is a prototype that emphasizes “suggested” diets rather than “recommended” ones. To progress to our goal of a dietary recommendation app, improved ranking confidence is required. Another limitation is that this approach is tailored to conditions that are caused by aberrant expression or activity of one or a small number of key risk genes. Polygenic diseases would be difficult to treat this way due to the need for many modulating dietary agents. Nonetheless, many conditions fit this “small number of key risk genes” description as evidenced by the many single genes targeted by pharmaceutical drugs. That said, it is important to emphasize that nutrition should not be a replacement to necessary and rational pharmacological treatment. It is also important to note that in addition to increasing the intake of healthy foods, app users should control the consumption of unhealthy and pervasive hyper-palatable foods including saturated fat or refined carbohydrates. Relative to this, it is worth noting that while our approach is skewed toward healthy diet due to all the associated “general” health benefits, even unhealthy diets modulate gene expression and in some cases, could be beneficial by modulating the expression of key disease-associated genes in the desired direction.

In conclusion, an interactive dietary guide app prototype has been constructed towards translating our DRGT strategy into an innovative, low-cost, healthy, and readily translatable public resource to improve health. To maximize the potential effectiveness of food suggestions, the app emphasizes mined public database data using human whole food ingestion, and is intended to eventually aid patients, healthcare providers, community and researchers in treating and prevent numerous health conditions. To underscore its utility, user scenarios from physician or patient and researcher perspectives are included. Our intent is that the prototype app presented in this paper is a valuable first step that sets the stage for its future expansion and development through (1) the continued addition of future diet and gene expression study data, and (2) the addition of data from clinical studies on specific foods targeting specific key disease genes carried out as guided by our app. In addition to the need of these two future developments, and depending upon future technology and cost, (3) successful future implementation of our app for treating pathologies for a given individual will most preferably include that individual assess their specific response to a healthy diet suggestion from our app; i.e., not all individuals will respond to diet the same way as our summarized mined data.

## Data availability statement

The original contributions presented in the study are included in the article/supplementary material, further inquiries can be directed to the corresponding author.

## Author contributions

Author contributions to the manuscript include web design and construction and data preparation by MF, JC, and ZX. Supervision of the web design and construction and data preparation by JE and KB. Dietary study data mining by VS. Data mining, creation of the dietary rational gene targeting strategy, dietary study data compilation, and assisting supervision of the web design and construction by DC. All authors contributed to the article and approved the submitted version.

## Funding

This work was supported by the Bender Scientific Fund, the Wildermuth Foundation, and the Institute for Data Explorations and Applications at Rensselaer Polytechnic Institute.

## Conflict of interest

The authors declare that the research was conducted in the absence of any commercial or financial relationships that could be construed as a potential conflict of interest.

## Publisher’s note

All claims expressed in this article are solely those of the authors and do not necessarily represent those of their affiliated organizations, or those of the publisher, the editors and the reviewers. Any product that may be evaluated in this article, or claim that may be made by its manufacturer, is not guaranteed or endorsed by the publisher.
